# Opioid-Induced Nausea Involves a Vestibular Problem Preventable by Head-Rest

**DOI:** 10.1371/journal.pone.0135263

**Published:** 2015-08-27

**Authors:** Nadine Lehnen, Fabian Heuser, Murat Sağlam, Christian M. Schulz, Klaus J. Wagner, Masakatsu Taki, Eberhard F. Kochs, Klaus Jahn, Thomas Brandt, Stefan Glasauer, Erich Schneider

**Affiliations:** 1 Centre for Sensorimotor Research, Munich University Hospital, Munich, Germany; 2 German Centre for Vertigo and Balance Disorders, Munich University Hospital, Munich, Germany; 3 Department of Neurology, Munich University Hospital, Munich, Germany; 4 Department of Anaesthesiology, Klinikum rechts der Isar, Technische Universität München, Munich, Germany; 5 Department of Biomedical Engineering, Faculty of Engineering and Architecture, Gediz University, Izmir, Turkey; 6 Department of Otolaryngology-HNS, Kyoto Prefectural University of Medicine, Kyoto, Japan; 7 Schön Klinik Bad Aibling, Bad Aibling, Germany; 8 Institute for Clinical Neurosciences, Munich University Hospital, Munich, Germany; 9 Institute for Medical Technology, Brandenburg Institute of Technology, Cottbus-Senftenberg, Germany; Tokyo Metropolitan Institute of Medical Science, JAPAN

## Abstract

**Background and Aims:**

Opioids are indispensable for pain treatment but may cause serious nausea and vomiting. The mechanism leading to these complications is not clear. We investigated whether an opioid effect on the vestibular system resulting in corrupt head motion sensation is causative and, consequently, whether head-rest prevents nausea.

**Methods:**

Thirty-six healthy men (26.6±4.3 years) received an opioid remifentanil infusion (45 min, 0.15 μg/kg/min). Outcome measures were the vestibulo-ocular reflex (VOR) gain determined by video-head-impulse-testing, and nausea. The first experiment (n = 10) assessed outcome measures at rest and after a series of five 1-Hz forward and backward head-trunk movements during one-time remifentanil administration. The second experiment (n = 10) determined outcome measures on two days in a controlled crossover design: (1) without movement and (2) with a series of five 1-Hz forward and backward head-trunk bends 30 min after remifentanil start. Nausea was psychophysically quantified (scale from 0 to 10). The third controlled crossover experiment (n = 16) assessed nausea (1) without movement and (2) with head movement; isolated head movements consisting of the three axes of rotation (pitch, roll, yaw) were imposed 20 times at a frequency of 1 Hz in a random, unpredictable order of each of the three axes. All movements were applied manually, passively with amplitudes of about ± 45 degrees.

**Results:**

The VOR gain decreased during remifentanil administration (p<0.001), averaging 0.92±0.05 (mean±standard deviation) before, 0.60±0.12 with, and 0.91±0.05 after infusion. The average half-life of VOR recovery was 5.3±2.4 min. 32/36 subjects had no nausea at rest (nausea scale 0.00/0.00 median/interquartile range). Head-trunk and isolated head movement triggered nausea in 64% (p<0.01) with no difference between head-trunk and isolated head movements (nausea scale 4.00/7.25 and 1.00/4.5, respectively).

**Conclusions:**

Remifentanil reversibly decreases VOR gain at a half-life reflecting the drug’s pharmacokinetics. We suggest that the decrease in VOR gain leads to a perceptual mismatch of multisensory input with the applied head movement, which results in nausea, and that, consequently, vigorous head movements should be avoided to prevent opioid-induced nausea.

## Introduction

Opioids are indispensable in the treatment of moderate to severe pain [1]. They are components of the number one oral prescription drugs in the US [2] and are applied several million times a year during general anaesthesia [3].

Opioid use can be complicated by the common adverse reactions of nausea and vomiting [4], occurring in a third of all patients treated with morphine equivalents [5]. The incidence of postoperative nausea and vomiting (PONV)–with opioids as a key causative factor [6]–is approximately 20–30% in the general population [7] and reaches up to 80% in PONV high-risk patients [6].

Opioid-induced nausea and vomiting is a significant threat: Patients rank it the most distressing non-life-threatening side effect [1] and consider vomiting the most undesirable complication after surgery [8]. They are willing to spend their own money [9] and even accept pain [8] in order to avoid it. Opioid-induced nausea and vomiting is a significant factor in complications such as pulmonary aspiration, dehydration, and electrolyte imbalance. Its treatment costs several million dollar a year [10], frequently delaying discharge and leading to unexpected hospital admissions after ambulatory surgery [11].

How opioids cause nausea and vomiting is still under debate [7]. It has been suggested that they have a direct effect on the chemoreceptor trigger zone in the area postrema or on the vomiting centre in the brainstem [7]. In addition, opioid-induced nausea seems to be triggered by head motion and avoided by rest [12]. This is why the vestibular system, which senses head motion, was thought to contribute [6]. But while opioid-receptors can be found within peripheral [13, 14] and central [15] vestibular structures, tests of vestibular function with opioid administration (e.g., caloric irrigation, galvanic stimulation, and active VOR-testing) were inconclusive since both a hyper- [16] and hypo- [12, 16–18] excitability were found.

We investigated the vestibulo-ocular reflex function under μ-receptor agonist infusion and tested the influence of movement on the incidence of opioid-induced nausea.

## Material and Methods

### Subjects

Thirty-six healthy men (ten for experiment 1, aged 28.1±6.3 years (mean±standard deviation), ten for experiment 2, aged 26.0±3.2 years, 16 for experiment 3, aged 26.1±3.2 years) took part in the study that was approved by the Ethics Committee of the Technische Universität München and in accordance with the Declaration of Helsinki. Subjects gave their written informed consent and were free to withdraw from the experiment at any time. They were financially compensated for taking part in the study. Subjects had no history of balance disorders and were not taking any medication (explicitly no opioids). They experienced no nausea or vomiting when being bent forward and backward passively five times at a frequency of 1 Hz (about ± 45 degrees, head-trunk movements) or after 20 head-bends for each of the three axes of rotation (pitch, roll, yaw) with a frequency of 1 Hz (about ± 45 degrees, isolated head movement) at inclusion to the study (without any drug administration).

The simplified risk score for predicting PONV [6], chosen because of its ubiquitous use in anaesthesiology, was 28±9%, 35±8% and 38±8% (mean±standard deviation) in the subjects of experiments 1 (n = 10), 2 (n = 10) and 3 (n = 16), respectively. According to this score, 35% of all subjects (13/36), on average, were expected to experience PONV. Motion sickness susceptibility (assessed by the Motion Sickness Susceptibility Questionnaire, MSSQ-Short [19]) was 4.8±5.0, 7.9±6.1 and 4.2±5.3 respectively. The MSSQ was used in addition to the PONV score to make sure that groups had comparable motion sickness susceptibility in addition to having comparable PONV risk. Subjects showed no abnormalities on a standard neurological exam. They had fasted for 6 hours and refrained from alcohol and smoking for more than 24 hours before the tests.

### Opioid administration

Remifentanil was administered continuously through a cubital vein at a rate of 0.15 μg/kg/min. It was chosen over other opioids because of its well-known pharmacokinetic characteristics of a steady-state plasma level after a short time of continuous intravenous administration (90% after 17 min) and a context-sensitive half-life time of 3.7 min after stopping the drip [20]. Standard monitoring was applied using ECG, non-invasive blood pressure and pulse oxymetry.

### Vestibular testing

Video-head-impulse-testing was used to assess horizontal rotational VOR function as previously described [21]. Briefly, passive high-acceleration (2000–6000°/s^2^), small amplitude (10–20°) head rotations to the right and left in the planes of the horizontal semicircular canals were applied by an experienced examiner while subjects fixated a target 2.50 meter away (standard head impulse testing [22]). During head impulse testing, eye movements were measured by video-oculography, head movements by integrated 6-degree-of-freedom inertial sensors (EyeSeeCam [21]). Eye and head movement data were sampled at a rate of 220 Hz. The gain of the VOR was determined for each head impulse as the ratio of the median of eye and head velocity between 55 and 65 ms after head movement start. Data for right and left head rotations were pooled.

### Assessment of nausea

#### Experiment 1

Subjects were asked if they experienced any nausea. The occurrence of vomiting was noted. Nausea and vomiting were assessed before starting the remifentanil drip, and after 30 min and 45 min of remifentanil infusion at a rate of 0.15 μg/kg/min.

#### Experiment 2 and 3

Nausea (maximum experienced for the relevant time period) was quantified psychophysically in arbitrary values on a numerical scale from 0 (“everything okay”) to 10 (“vomiting”). The 11-point scale was chosen in analogy to Apfel et al. [23]. Quantification occurred before starting the remifentanil drip and after 45 min of remifentanil infusion at a rate of 0.15 μg/kg/min.

Nausea was always assessed before video-head-impulse testing (experiments 1 and 2).

### Experimental paradigms

**Fig 1:**
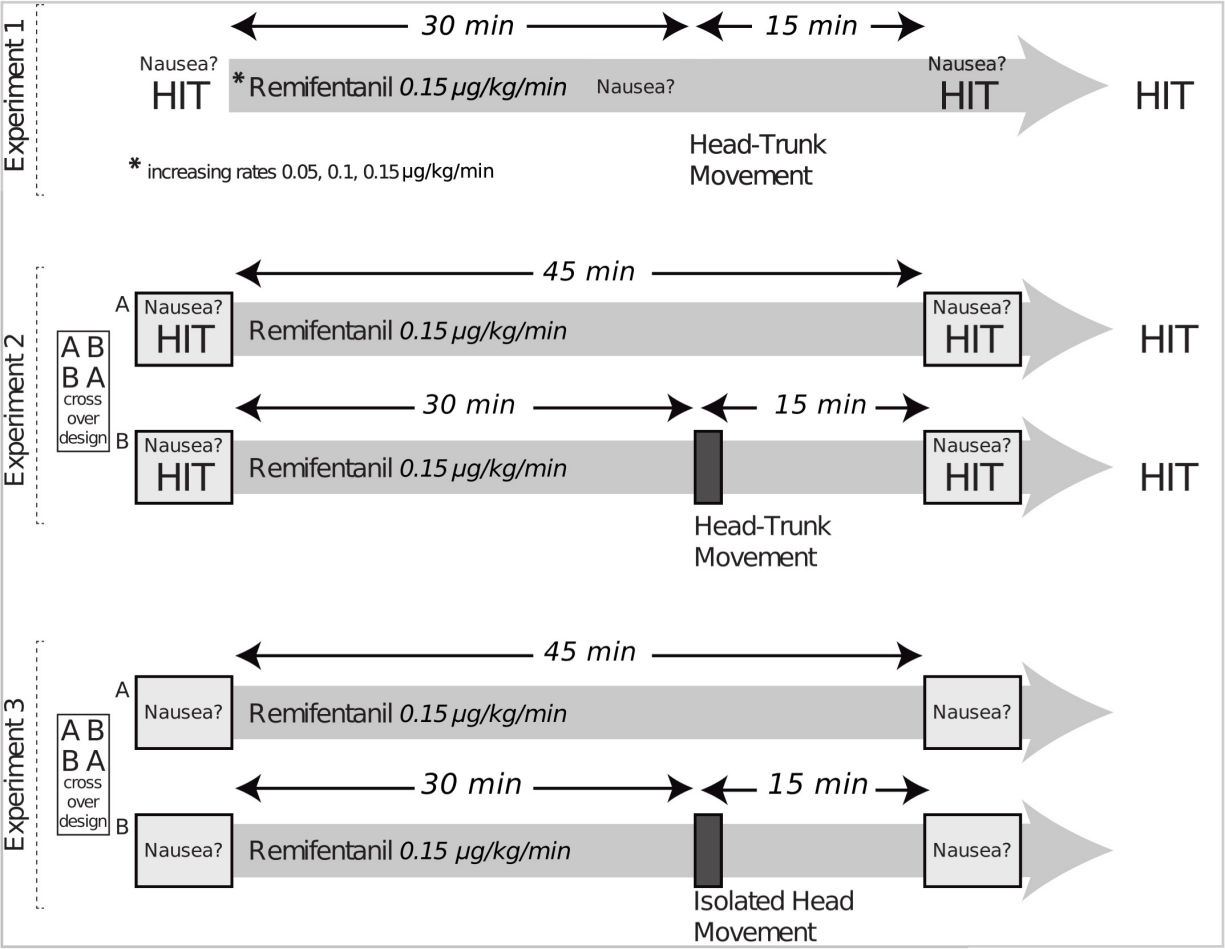
Experimental design. Experiment 1 (ten subjects): Subjects lay in a semirecumbent position during continuous intravenous administration of remifentanil (increasing rates of 0.05 μg/kg/min, 0.10 μg/kg/min and 0.15 μg/kg/min). After 30 min at 0.15 μg/kg/min, subjects were manually passively bent forward and backward five times at a frequency of 1 Hz (head-trunk movement, about ± 45 degrees). Nausea assessment was performed before, after 30 min before movement and after 45 min of continuous administration of remifentanil at 0.15 μg/kg/min. The head-impulse test (HIT) of vestibular function was performed before and 45 min after initiating remifentanil at 0.15 μg/kg/min. Experiment 2 (ten subjects): There were two paradigms: in the paradigm “rest” (A), subjects lay motionless in a semirecumbent position during 45 min of continuous intravenous administration of remifentanil. Nausea assessment (numerical scale from 0–10) and head-impulse testing (HIT) of vestibular function were performed before and 45 min after initiating remifentanil. The paradigm “head-trunk move” (B) was identical to the paradigm “rest” except for one additional intervention: after 30 min, subjects were manually passively bent forward and backward five times at a frequency of 1 Hz (“head-trunk move”, amplitude about ± 45 degrees). In a crossover design, subjects were tested twice with at least 1 day washout between measurements. Five subjects were first tested with paradigm “rest” (A) and then with paradigm “head-trunk move” (B), and vice versa. Experiment 3 (sixteen subjects): The crossover design was the same as in experiment 2 but instead of the paradigm “head-trunk move” a paradigm “head move” was done (twenty isolated head movements for each of the three axes of rotation (pitch, roll, yaw) performed randomly and unpredictable with a frequency of 1 Hz). Eight subjects were first tested with paradigm “rest” (A) and then with paradigm “head move” (B), and vice versa.

Experiment 1 was designed to test the effect of remifentanil. Experiment 2 had a crossover design to further verify the motion-dependency and, in particular, to exclude the possibility of a simple time effect, i.e., that nausea and vomiting would have occurred after prolonged remifentanil exposure even without movement. To verify that head motion alone, sensed by the vestibular system, leads to nausea during opioid-use, experiment 3 was designed as a cross-over design with the paradigm “rest” being compared to a paradigm “head move” consisting of head movement alone (trunk stationary). Graphical explanations of all experiments are in Fig 1.

#### Experiment 1

Remifentanil was continuously administered with increasing rates of 0.05 μg/kg/min, 0.10 μg/kg/min and 0.15 μg/kg/min while subjects lay in a semirecumbent position (total time of administration at 0.15 μg/kg/min: 45 min). The occurrence of nausea and vomiting was documented before starting the drip and during remifentanil administration at 0.15 μg/kg/min at rest and after consecutive passive ± 45 degree head-trunk movements, which were applied manually (forward and backward five times at a frequency of 1 Hz). Head-impulse testing was performed before and during continuous administration of remifentanil, and, as far as nausea and vomiting permitted, continuously during the 30 min after stopping remifentanil (9/10 subjects).

#### Experiment 2

The ten subjects were divided into two equal subgroups with no significant differences in age (25.6±3.1 years and 26.4±3.5 years, mean±standard deviation; t-test, p = 0.71), MSSQ (8.9±7.6 points, 6.9±4.8 points; p = 0.63) and PONV-risk (39±0%, 32±10%; p = 0.14). In a crossover design, each subject was tested twice with at least a day washout period between the measurements. Five subjects were first tested with a paradigm “rest” and then with a paradigm “head-trunk move”, and vice versa. In the paradigm “rest”, subjects rested in a semirecumbent position during 45 min of continuous intravenous administration of remifentanil (0.15 μg/kg/min). Nausea assessment, head-impulse testing, and a clinical oculomotor exam were performed before and 45 min after initiating remifentanil. The paradigm “head-trunk move” was the same as the paradigm “rest” except for one additional intervention: after 30 min, subjects’ head-trunk was manually and passively bent forward and backward five times at a frequency of 1 Hz with a motion range of about ± 45 degrees. In both paradigms, subjects were continuously encouraged to report any opioid effects they experienced. At the end of each experiment, subjects were asked to name the opioid effect they experienced as most unpleasant.

#### Experiment 3

The sixteen subjects were divided into two equal subgroups with no significant differences in age (26.2±2.3 years and 25.9±4.1 years, mean±standard deviation; t-test, p = 0.82), MSSQ (2.9±3.3 points, 5.4±6.8 points; p = 0.37) and PONV-risk (37±6%, 40±11%; p = 0.54). The crossover design was the same as in experiment 2, but instead of the paradigm “head-trunk move” a paradigm “head move” was employed; passive, isolated head movements consisting of the three axes of rotation (pitch, roll, yaw) with a motion range of approximately ± 45 degrees were imposed manually 20 times at a frequency of 1 Hz in a random, unpredictable order of each of the three axes.

Note that, due to practicability in the operating room, the head was moved differently in experiment 2 (movement together with trunk) and 3 (isolated head movement).

### Statistical analysis

For experiment 1, differences in VOR gain within the conditions (factors: before, with, and after remifentanil) were assessed by a repeated-measures analysis of variance (ANOVA). The half-life time of VOR recovery was determined from the continuous head-impulse testing after stopping the drip. An exponential function was fitted to the VOR gain values by means of least square fitting (function fminsearch, MATLAB, Mathworks, Natick, MA, USA). The quality of individual fits was expressed by the R^2^ value. The mean R^2^ was calculated after Fisher’s z-transformation.

For experiment 2, ANOVA was used to determine differences in VOR gain within the conditions and paradigms (factors: “rest” and “head-trunk move”) and between the subgroups of the crossover design. A Bonferroni corrected post-hoc test was used for pairwise comparison. Differences in nausea scale ratings between the subgroups were assessed with an independent samples Mann-Whitney U test, differences within the paradigms (“rest” and “head-trunk move”) with a related samples Friedman’s ANOVA by ranks.

For experiment 3, differences in nausea scale ratings between the subgroups were assessed with an independent samples Mann-Whitney U test, differences within the paradigms (“rest” and “head move”) with a related samples Friedman’s ANOVA by ranks.

The difference in nausea rating between the “rest” and “head-trunk/head move” conditions was computed for experiments 2 and 3. Independent samples Mann-Whitney U test was used to assess whether this difference is the same in experiments 2 and 3, i.e. in the “head-trunk move” and “head move” conditions.

Kolmogorov-Smirnov test was used to examine normal distribution of VOR gain values, Spearman correlation analysis to examine correlation between a decrease in the VOR gain and the score of motion-induced nausea, and Fisher’s exact test to compare the actual and expected incidences of nausea.

In all statistical analyses, significance levels were p<0.05. All data are fully available without restriction.

## Results

### Vestibular function

**Fig 2:**
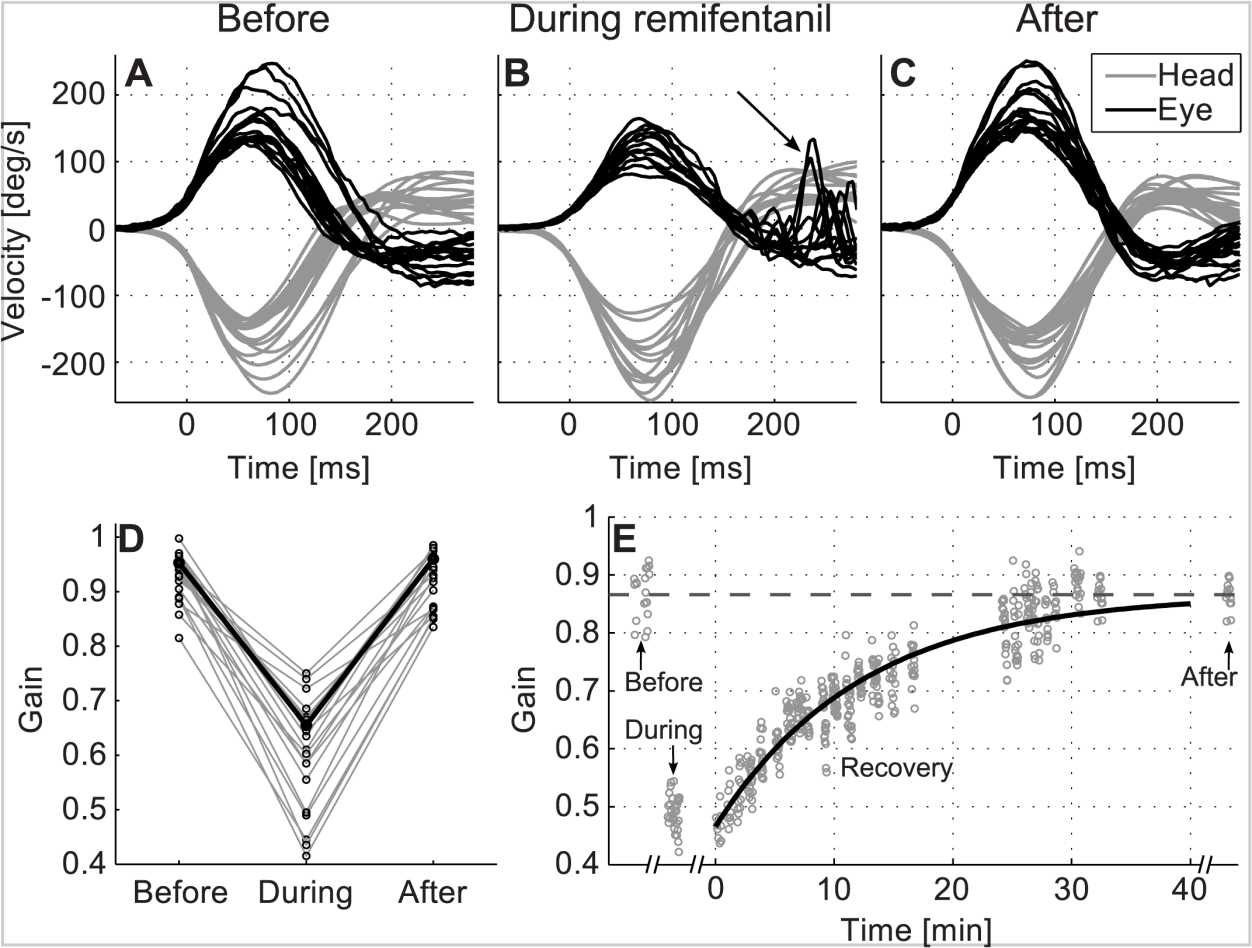
Remifentanil effects on the head impulse gain of the vestibulo-ocular reflex. A-C: Eye (black) in response to head (grey) velocity traces during head-impulse testing in one subject (left (sign-inverted) and right side pooled) before (A), during (B) and after (C) continuous remifentanil administration. Before remifentanil, eye velocity was exactly opposite to head velocity. The vestibulo-ocular reflex (VOR) was intact. With remifentanil administration (B), eye velocity did not match head velocity. The subject used catch up saccades (arrow) to compensate for the deficient VOR. Thirty minutes after stopping remifentanil (C), the VOR was intact again. D: Mean head impulse gain of the VOR for each of the 20 subjects of experiments 1 and 2 (individual circles) before (left), during (middle) and after (right) remifentanil administration. Before averaging, the VOR gain was calculated for each head impulse as the ratio of the median of eye and head velocity between 55 and 65 ms after head movement start. The gain decreased with remifentanil in each subject and returned to baseline afterwards. Black line denotes the subject in A, B, and C. E: Recovery of VOR gain after stopping remifentanil infusion. Circles represent the VOR gain during head-impulse testing of one subject before, during and in the 30 minutes after stopping remifentanil. The gain recovery after stopping the infusion follows an exponential increase reflecting the half-life time of the drug. In this subject, the half-life time was 8.5 min (R^2^ = 0.98).

Fig 2 shows eye (black) and head (grey) velocity traces during head impulse testing in one exemplary subject before (A), during (B) and after (C) continuous intravenous administration of remifentanil. Before remifentanil, the VOR was intact: the eyes exactly mirrored the head movements and kept gaze stable on the target (A). With remifentanil administration, the gain of the VOR significantly decreased: the compensatory eye velocity did not match head velocity (B), the eyes moved together with the head and the subject used catch-up saccades (arrow) to re-fixate the target. Thirty minutes after stopping the remifentanil drip, the VOR was intact again (C).

In all subjects, the VOR function consistently decreased with remifentanil (Fig 2D). ANOVA revealed that remifentanil had a substantial effect on the VOR gain (p<0.001, experiments 1 and 2); this was independent of the subgroup (p = 0.39) and paradigm (“rest” or “head-trunk move”, p = 0.53) of experiment 2. The average gain of the VOR was 0.92±0.05 before remifentanil (mean±standard deviation); it decreased to 0.60±0.12 with remifentanil and returned to base level after remifentanil stopped (0.91±0.05, no difference from base level, p = 0.86 and p = 0.53 in experiment 1 and 2, respectively). VOR recovery after stopping remifentanil followed an exponential increase with an average half-life time of 5.3±2.4 min (R^2^ = 0.94±0.03, mean±standard deviation, see Fig 2E for an example).

### Nausea and vomiting

**Fig 3:**
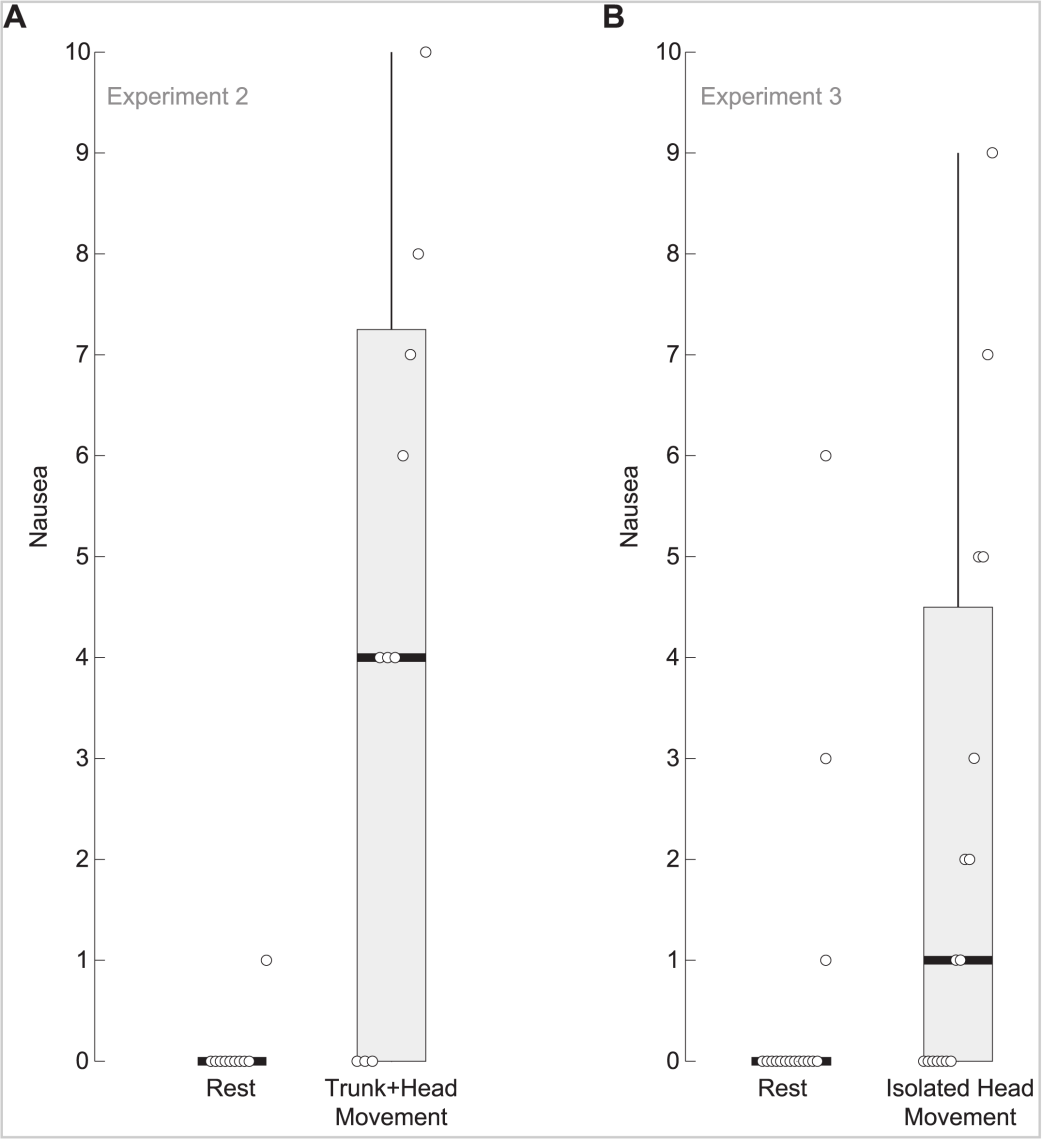
Effects of head-trunk and isolated head movement on nausea with remifentanil administration (cross-over design). Median (horizontal bold line) and interquartile range (box) of nausea values quantified on a numerical scale from 0 (“everything okay”) to 10 (“vomiting”) in paradigms “rest” (left) and “head-trunk/head move” (right). Data of each subject is shown by a white dot. A: Experiment 2, head-trunk movement led to a marked increase in nausea (p = 0.008, related samples Friedman’s ANOVA by ranks). B: Experiment 3, isolated head movement led to a marked increase in nausea (p = 0.005, related samples Friedman’s ANOVA by ranks). The difference in nausea rating between the “rest” and “head-trunk/head move” conditions were not different for the groups who performed head-trunk or isolated head movement (Independent samples Mann-Whitney U test, p = 0.068).

In experiment 1, none of the subjects reported nausea while lying still. After movement, seven subjects reported nausea; three of them vomited. This indicates a motion-dependency of nausea and vomiting with remifentanil use. Experiment 2 was designed to further verify this motion-dependency and, in particular, to exclude the possibility of a simple time effect, i.e., that nausea and vomiting would have occurred after prolonged remifentanil exposure even without movement. In the “rest” paradigm, one subject out of ten reported “a touch” of nausea (nausea scale 1) after 45 min of continuous remifentanil administration. In the “head-trunk move” paradigm, seven subjects reported nausea after movement with nausea scale values ranging from 4 to 10 (Table 1, first five subjects are subgroup 1). There was no difference in nausea scale values between the two subgroups of the crossover design (independent samples Mann-Whitney U test, p = 0.69 for the “rest” condition, p = 0.42 for the “head-trunk move” condition) and therefore no conditioning or learning effect. Fig 3A shows pooled nausea scale values for both subgroups: they were 0.00/0.00 (median/interquartile range) without movement and 4.00/7.25 with movement. This represents a substantial difference (related samples Friedman’s ANOVA by ranks, p = 0.008) and shows that nausea and vomiting during remifentanil administration were triggered by motion.

**Table 1:** Unpleasantness of remifentanil effects (experiment 2).

Subject	Paradigm „rest“		Paradigm „move“	
	Nausea scale	Most unpleasant opioid effect	Nausea scale	Most unpleasant opioid effect
1	0	Fixating difficulties	0	Swallowing difficulties
2	0	Slow breathing	8	Nausea
3	0	Feeling heavy	0	Itching
4	0	Itching	7	Nausea
5	0	Itching	0	Itching
6	0	Fixating difficulties	4	Nausea
7	0	Swallowing difficulties	4	Nausea
8	0	Feeling heavy	6	Nausea
9	0	Itching	4	Nausea
10	1	Swallowing difficulties	10	Nausea

There was no significant correlation between gain-drop (VOR-gain-before-remifentanil minus VOR-gain-during-remifentanil) and nausea rating in the head-trunk move condition (experiment 2, Spearman correlation, r = -0.20 and p = 0.58). Also, independent samples t-test did not reveal a difference in gain-drop between the patients who vomited (nausea score 10) and those who had no nausea (p = 0.67, experiments 1 and 2, t-test for normally distributed gain values according to Kolmogorov-Smirnov test, p = 0.2).

To verify that isolated head motion, sensed by the vestibular system, can trigger nausea during opioid-use, experiment 3 was designed as a cross-over design with the paradigm “rest” being compared to a paradigm “head move” consisting of head movement alone (trunk stationary). Fig 3B shows nausea scale values for “rest” and “head move” conditions. In the “rest” paradigm, three out of the sixteen subjects reported nausea (nausea scales 1, 3 and 6). During the “head move” paradigm, nine subjects reported nausea after movement with nausea scale values ranging from 2 to 9. There was no difference in nausea scale values between the two subgroups of the crossover design (independent samples Mann-Whitney U test, p = 0.064 for the “rest” condition, p = 0.784 for the “head move” condition) and therefore no conditioning or learning effect. Pooled nausea scale values for both subgroups were 0.00/0.00 (median/interquartile range) without movement and 1.00/4.5 with movement. This represents a substantial difference (related samples Friedman’s ANOVA by ranks, p = 0.005) and shows that nausea and vomiting during remifentanil administration were triggered by isolated head motion.

The difference in nausea ratings between the “rest” and “head-trunk/head move” conditions were not different for the groups who performed head-trunk (experiment 2) or isolated head movement (experiment 3; independent samples Mann-Whitney U test, p = 0.068).

Overall, the nausea incidence during remifentanil infusion at rest in our study (4/36 or 11%) was lower than the PONV incidence expected according to the Apfel score ([6], 35% or 13/36, p = 0.025 by Fischer’s exact test); the nausea incidence with motion during remifentanil infusion (23/36 or 64%) was higher than that expected from this score (p = 0.033, Fischer’s exact test).

### Ranking: unpleasantness of opioid effects

All subjects from experiment 2 who experienced nausea greater than 1 on the nausea scale, i.e., all subjects from the “head-trunk move” paradigm that experienced nausea, rated it the most unpleasant remifentanil effect (Table 1). Those without any relevant nausea rated various other opioid effects as the most unpleasant: itching, difficulties swallowing, feeling heavy, difficulties fixating, and slow breathing. The rating varied within the same subject between the conditions.

### Oculomotor findings

Remifentanil induced downbeat nystagmus in all subjects. In some subjects, there were additional cerebellar oculomotor signs such as saccadic smooth pursuit and gaze-evoked nystagmus. All oculomotor findings ceased after ending the remifentanil drip.

## Discussion

Our findings indicate that remifentanil had an effect on the vestibular system since the VOR gain decreased in all subjects, experiments and conditions assessed. The half-life time of VOR gain recovery after stopping remifentanil infusion (5.3±2.4 min, mean±standard deviation) reflects the pharmacokinetics of remifentanil [20]. Nausea during remifentanil administration was triggered by head movement in the majority of subjects. Four of the thirty-six subjects also experienced mild to moderate nausea at rest.

The remifentanil effect on the vestibular system agrees with previous observations that noted a decrease in caloric response with morphine [12], a diminished active VOR with pethidine and fentanyl administration [17], and of passive VOR and dynamic vision with remifentanil [18] and vestibular dysfunction with heroin abuse [24].

Opioid effects on receptors in the VOR-three-neuron arc [13, 14] and in the cerebellum [15] could mediate the changes in vestibular-ocular motor function (VOR gain). The additional oculomotor findings, such as gaze-evoked nystagmus, saccadic smooth pursuit and, in particular, downbeat-nystagmus, point to an involvement of the cerebellum [17].

When the VOR gain was decreased, head motion (either as part of head-trunk motion or isolated) triggered nausea and vomiting. The incidence of nausea at rest during remifentanil infusion (4/36 or 11%), however, was significantly lower than that expected from the Apfel score ([6], 35% or 13/36). This is in line with observations that motion worsens the incidence and severity of PONV [6] and indicates that, in addition to possible direct opioid effects on the chemoreceptor trigger zone in the area postrema or the vomiting centre in the brainstem [7], which lead to nausea at rest, there is a head-movement triggered pathomechanism. With a motion-induced nausea incidence of 23/36 or 64%, which is significantly higher than the incidence expected from the Apfel score ([6], 13/36 or 35%), the present study overestimates the motion-induced mechanism, possibly because the stimuli were more vigorous than the ones patients commonly experience perioperatively. Importantly, whereas all our subjects who experienced nausea with movement rated it the worst side effect, which corresponds to patients ranking nausea the most distressing non-life-threatening side effect [1] and consider vomiting the most undesirable complication after surgery [8], this was not the case for nausea at rest.

The discordance between movement information from different sensors (e.g., visual, proprioceptive, vestibular) or between expected and real sensory information is thought to cause nausea and vomiting of motion sickness [25–30]. Similarly, movement-induced nausea during opioid administration could be due to a mismatch either within opioid-corrupted vestibular input or between vestibular input and that of other sensors (e.g. vision, proprioception). This would become apparent during movement generating vestibular input, i.e., head movement. As in motion sickness, nausea and vomiting would then result from mismatched input to the vomiting centre in the lateral reticular formation in the medulla oblongata and the chemoreceptor trigger zone in the area postrema. Perception has been shown to correlate with VOR function [31–33]. As this study demonstrates a corrupted VOR response with opioid administration, nausea could result from the corrupted perceptual response.

In our experiments, remifentanil itself was emetogenic only in some subjects at rest, while being so in a majority of subjects during movements. Further, the head motion itself induced nausea in none of our subjects before remifentanil decreased VOR gains, whereas it induced nausea in a majority of subjects after remifentanil decreased VOR gains. These findings clearly indicated that remifentanil induced vestibular dysfunction, evaluated as decreases in VOR gains, and that vigorous head motions manifested opioid-induced motion sickness though such vestibular dysfunction, thereby exacerbating remifentanil-induced nausea. Although we could not find any significant correlation between VOR gain drops and nausea scores, this may simply reflect the possibility that the mismatch that triggered nausea can be independent of the amount of gain change—similarly to motion sickness where symptom strength poorly correlates with that of the provoking stimulus [34].

Due to practicability in the operation room, the head was moved differently in experiment 3 as opposed to the head-trunk movements in experiment 2. This could explain the (non-significant but due to the small numbers maybe critical) trend that a larger proportion of subjects did not suffer from nausea at all when the head only was moved. However, importantly, experiment 3 was designed to analyse whether head motion alone leads to nausea during opioid-use, which it does. So, even assuming that there was a difference in nausea ratings between experiments 2 and 3 and that this was owing to the fact that the head was moved differently, the conclusion that head movement alone can greatly exacerbate nausea during opioid use would still be valid.

The relative contribution of the movement-triggered pathomechanism and other direct opioid effects is unknown from our study. The motion sickness part of the pathomechanism is in line with several observations about PONV:

It is well known that once motion sickness is elicited, it outlasts the precipitating stimuli for hours or up to the entire day [35]. Similarly, the decomposition of opioids and VOR recovery do not necessarily terminate nausea in afflicted subjects. This could explain why ultra short-acting opioid remifentanil has been found to have a PONV incidence similar to that of longer acting fentanyl during the first postoperative 24 hours [23].

Only a few risk factors are independent predictors for PONV: a history of motion sickness or PONV, female sex, non-smoking, and the use of postoperative opioids [6]. These risk factors are in line with our suggestion that head motion during transient impairment of vestibular function plays a role in opioid-induced nausea: if opioid-induced nausea and motion sickness have a common pathomechanism, it is evident why motion sickness susceptibility also predisposes to opioid-induced nausea and vomiting [6] and why women, who are significantly more susceptible to motion sickness [29, 36] also have a higher PONV risk [6]. Interestingly, both motion sickness and opioid-induced nausea also are more common in the 6–10 year age group [37, 38] and decrease with age [39, 40]. Tolerance to motion sickness is increased by short-term nicotine withdrawal [41]–as is routinely practiced perioperatively. We suggest that as non-smokers cannot benefit from this effect they have a higher PONV risk [6].

Opioid effects such as cognitive slowing and sleepiness are not mediated by the vestibular system. They can, however, also lead to a feeling of light-headedness at rest, but not to nausea or vomiting. In our study, all subjects (experiment 2) who experienced relevant nausea rated it the most unpleasant remifentanil effect. When no relevant nausea was present, the most unpleasant side effects varied, even between the paradigms (“head-trunk/head move” and “rest”) in the same subject. This, again, underlines the unpleasantness of nausea and vomiting compared to other adverse effects such as cognitive slowing or light-headedness.

Head-rest as a preventive measure is feasible particularly in the perioperative setting with short opioid treatments during general anaesthesia where vigorous movements applied when patients are transferred out of the sterile area of the operation room, for example, can easily be avoided during the time opioids are effective. Further studies should address which sensory mismatch, e.g. visuo-vestibular or intra-vestibular, is relevant and, consequently whether in addition to head-rest, darkness could help prevent opioid-induced nausea.

In conclusion, we showed that the μ-agonist remifentanil affects the vestibular function in its pharmacokinetics-dependent manner and that vigorous head motion can extremely exacerbate nausea during opioid use. We suggest that the decrease in VOR gain causes an inter-sensory mismatch during head movements, which results in nausea and vomiting. This could be termed opioid-induced motion sickness. Consequently, vigorous head movements should be avoided to prevent opioid-induced nausea.
